# A multicenter randomized placebo controlled trial of rifampin to reduce pedal amputations for osteomyelitis in veterans with diabetes (VA INTREPID)

**DOI:** 10.1186/s12879-019-4751-3

**Published:** 2020-01-08

**Authors:** Mary T. Bessesen, Gheorghe Doros, Adam M. Henrie, Kelly M. Harrington, John A. Hermos, Robert A. Bonomo, Ryan E. Ferguson, Grant D. Huang, Sheldon T. Brown

**Affiliations:** 1 Department of Veterans Affairs Eastern Colorado Healthcare System, Denver, CO USA; 2 Division of Infectious Diseases, Department of Medicine, University of Colorado – Denver, Aurora, CO USA; 3 Massachusetts Veterans Epidemiology Research and Information Center, VA Boston Healthcare System, Boston, MA USA; 4 Department of Biostatistics, Boston University, Boston, MA USA; 5 Department of Veterans Affairs, Cooperative Studies Program Clinical Research Pharmacy Coordinating Center, Office of Research and Development, Albuquerque, NM USA; 6 Department of Psychiatry, Boston University School of Medicine, Boston, MA USA; 7 Department of Medicine, Boston University School of Medicine, Boston, MA USA; 8 Cleveland Department of Veterans Affairs Medical Center, Cleveland, OH USA; 9 Case Western Reserve University, Cleveland, OH USA; 10 Department of Epidemiology, Boston University School of Public Health, Boston, MA USA; 11 Department of Veterans Affairs, Cooperative Studies Program Central Office, Washington, DC USA; 12 James J. Peters VA Medical Center, New York, NY USA; 13 Department of Medicine, Icahn School of Medicine at Mt. Sinai, New York, NY USA

**Keywords:** Rifampin, Osteomyelitis, Diabetic foot, Amputation, Survival, Lower extremity, Diabetes mellitus, Double-blind, Clinical trial, Veterans

## Abstract

**Background:**

The prevalence of diabetes mellitus continues to inexorably rise in the United States and throughout the world. Lower limb amputations are a devastating comorbid complication of diabetes mellitus. Osteomyelitis increases the risk of amputation fourfold and commonly presages death.

Antimicrobial therapy for diabetic foot osteomyelitis (DFO) varies greatly, indicating that high quality data are needed to inform clinical decision making. Several small trials have indicated that the addition of rifampin to backbone antimicrobial regimens for osteomyelitis outside the setting of the diabetic foot results in 28 to 42% higher cure rates.

**Methods/design:**

This is a prospective, randomized, double-blind investigation of the addition of 6 weeks of rifampin, 600 mg daily, vs. matched placebo (riboflavin) to standard-of-care, backbone antimicrobial therapy for DFO. The study population are patients enrolled in Veteran Health Administration (VHA), ages ≥18 and ≤ 89 years with diabetes mellitus and definite or probable osteomyelitis of the foot for whom an extended course of oral or intravenous antibiotics is planned. The primary endpoint is amputation-free survival. The primary hypothesis is that using rifampin as adjunctive therapy will lower the hazard rate compared with the group that does not use rifampin as adjunctive therapy. The primary hypothesis will be tested by means of a two-sided log-rank test with a 5% significance level. The test has 90% power to detect a hazard ratio of 0.67 or lower with a total of 880 study participants followed on average for 1.8 years.

**Discussion:**

VA INTREPID will test if a rifampin-adjunctive antibiotic regimen increases amputation-free survival in patients seeking care in the VHA with DFO. A positive finding and its adoption by clinicians would reduce lower extremity amputations and their associated physical and emotional impact and reduce mortality for Veterans and for the general population with diabetic foot osteomyelitis. Given that rifampin-adjunctive regimens are currently employed for therapy for the majority of DFO cases in Europe, and only in a small minority of cases in the United States, the trial results will impact therapeutic decisions, even if the null hypothesis is not rejected.

**Trial registration:**

Registered January 6, 2017 at ClinicalTrials.gov, NCT03012529.

## Background

Diabetes mellitus prevalence is rising inexorably in the Unites States and globally. The 2011–2012 National Health and Nutritional Examination Survey (NHANES) data indicate that 14.3% of Americans have diabetes mellitus, and 38% have prediabetes [1]. Strategies for management of hyperglycemia have improved, but severe complications continue to impact survival and quality of life among diabetics. Lower extremity ulcerations with soft tissue and bone infections are common complications of diabetes mellitus with potentially devastating consequences. Osteomyelitis is found underlying 20% of all infected diabetic foot ulcers, and 60% of severe infections [2]. In a recent study, the risk of amputation was 14% in patients with infection limited to the soft tissue of the foot, and 60% in patients with osteomyelitis [3]. Therefore, control of bone infection is an important target in efforts to improve limb salvage in diabetics.

There is considerable variability in therapy prescribed for diabetic foot osteomyelitis (DFO) and there are few high-quality controlled trials of DFO to guide selection of antibiotic treatment regimens [4]. Adjunctive rifampin therapy is commonly employed in Europe, where 56 to 100% of practitioners select oral antimicrobial therapy with adjunctive rifampin for osteomyelitis [5] including DFO [6–9]. Use of rifampin for DFO in the United States is uncommon. The frequency of direct toxicity is similar for rifampin and other antibacterial agents used for bone infections [10]. The lack of randomized controlled data supporting rifampin therapy in DFO may contribute to U.S. physicians’ choice to omit rifampin therapy for DFO. A large controlled trial of adjunctive therapy for DFO has significant potential to impact practice if clinical benefit is confirmed and would also supply a wealth of data on DFO diagnostics, management and outcomes.

Rifampin has unique properties that make it an attractive adjunctive agent for DFO. It penetrates osteoblasts and retains antimicrobial activity within these cells [11]. Rifampin also penetrates biofilms and retains activity within them [12]. Adjunctive rifampin therapy has improved outcomes in several studies of osteomyelitis outside the setting of the diabetic foot [13, 14]. There are limited comparative data available on the impact of rifampin therapy of osteomyelitis in the setting of the diabetic foot. Effect sizes in randomized trials of rifampin for osteomyelitis not limited to the diabetic foot range from 28 to 42%.

Rifampin has broad spectrum activity against gram-positive organisms, which are the most common pathogens in DFO. *S. aureus* is the most common bacteria recovered from bone cultures in DFO. Other gram-positive organisms, including coagulase negative staphylococci and streptococci are recovered from 30 to 70% of cases [15]. Gram-negative organisms are found in a minority of cases of DFO. Clinical activity of rifampin against gram-negative pathogens has been observed in combination therapy of serious gram-negative infections that had failed other therapies [16, 17]. Antimicrobial activity from rifampin may consequently be seen in most cases of DFO.

In summary, rifampin’s broad antimicrobial spectrum, potent bactericidal activity, tissue penetration, and activity within biofilms, along with accumulating evidence from clinical trials in non-diabetic osteomyelitis and uncontrolled clinical experience in DFO make it attractive for formal study as an adjunctive therapy in DFO. A large pragmatic trial, enrolling patients with DFO without regard to culture results, will be feasible and will provide results that are generalizable to the broad population of DFO patients.

## Methods/study design

**Fig. 1:**
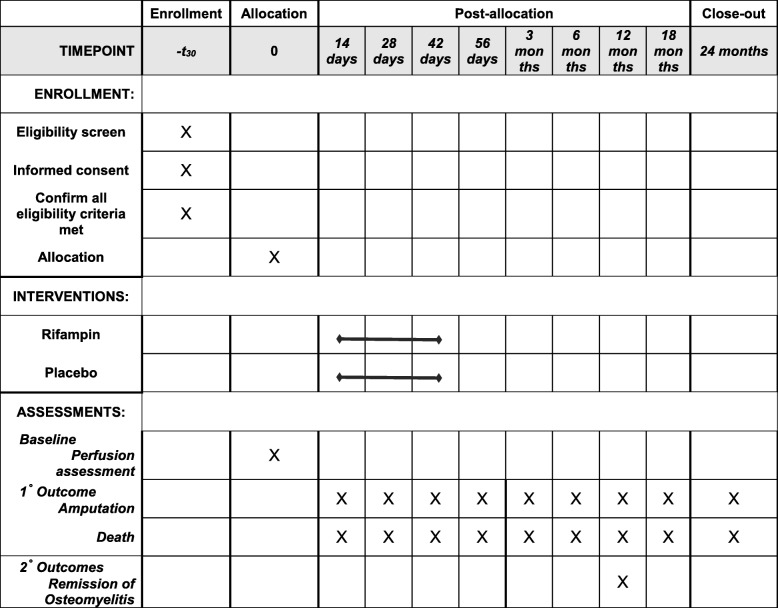
Schedule of Study Procedures

VA-INTREPID is a prospective, randomized, double-blind, placebo-controlled, investigation of a six-week course of adjunctive rifampin vs. adjunctive matched placebo (containing riboflavin) added to backbone antibacterial therapy for the treatment of definite or probable DFO, as defined by the International Working Group on the Diabetic Foot, summarized in Table 1 [18]. Backbone antibacterial therapy will be selected by the clinical treatment team and can be administered either intravenously or orally. The primary outcome measure is amputation-free survival. Amputation events include both below- and above-ankle amputations. Primary outcomes will be determined by systematic medical record review and through confirmatory research visits, phone calls and, as needed, information from non-VA providers. The secondary outcomes of complete wound epithelialization and remission of osteomyelitis will be determined by direct examination by the site investigators. Participants will have in person visits at baseline, 2, 4, and 6 weeks, 3 months, 6 months, and 12 months. The medical record will be reviewed up to 24 months to ascertain endpoints. Key secondary objectives are 1) to determine the differential effect of adding rifampin versus placebo to backbone antibiotic treatment on the time to each component of the primary endpoint, 2) to assess the heterogeneity of response to adjunctive rifampin treatment by specific subgroups, a) route of administration of backbone antibiotic therapy (IV vs. oral) b) baseline microbiological culture results (staphylococcal infections vs. non-staphylococcal infections vs. no culture) and c) baseline measures of vascular perfusion (toe pressure, TCpO2). Figure 1 describes the visit schedule and key procedures used throughout the study.

**Table 1:** Diagnostic criteria for DFO (adapted from Berendt et al., 2008 with permission)

Category	Post-test probability of osteomyelitis	Management advice	Criteria
Definite	> 90%	Treat for osteomyelitis	Bone sample with positive culture AND positive histology OR Purulence in bone found at surgery OR Atraumatically detached bone fragment removed from ulcer by podiatrist/surgeon OR Intraosseous abscess on MRI OR Any two probable criteria OR one probable and two possible criteria OR any four possible criteria below
Probable	51–90%	Consider treating	Visible cancellous bone in ulcer OR MRI showing bone edema with other signs of osteomyelitis OR Bone sample with positive culture but negative or absent histology OR Bone sample with positive histology but negative or absent culture OR Any two possible criteria below
Possible	10–50%	Treatment may be justified, but further investigation usually advised	Plain X-rays show cortical destruction OR MRI shows bone edema or cloaca OR Probe to bone positive or visible cortical bone OR ESR > 70 mm/hr. with no other plausible explanation OR Non-healing wound despite adequate offloading and perfusion for >6 weeks or ulcer of >2 weeks duration with clinical evidence of infection
Unlikely	< 10%	Usually no need for further investigation or treatment	No signs or symptoms of inflammation AND normal X-rays AND ulcer present for <2 weeks or absent AND any ulcer present is superficial OR Normal MRI OR Normal bone scan

### Study setting

VA-INTREPID is sponsored and conducted by the Department of Veterans Affairs (VA) Cooperative Studies Program (CSP), a research infrastructure that is dedicated to improving the healthcare of veterans through the conduct of clinical trials [19]. The Massachusetts Veterans Epidemiology Research and Information Center (MAVERIC) serves as the study’s CSP coordinating center (CSPCC), providing project management and statistical support, and the CSP Clinical Research Pharmacy Coordinating Center (CSPCRPCC) serves as the study’s drug distribution center, providing clinical trial materials and safety monitoring.

Study sites were selected based on caseload and investigator resources and are listed in Table 2. Patients between the ages of 18 and 90 years will be recruited from Podiatry, Primary Care, Surgery, Infectious Diseases and Endocrine clinics and from inpatient services at 28 Department of Veterans Affairs Medical Centers across the United States. Potential participants will also be identified from Radiology, Pathology and Surgery logs.

**Table 2:** Participating sites

Study Sites	Location
Study Chairs’ Offices	
James J. Peters VA Medical Center	Bronx, NY
VA Eastern Colorado Healthcare System	Denver, CO
Enrollment sites	
Atlanta VA Medical Center	Atlanta, GA
Bay Pines VA Healthcare System	Bay Pines, FL
Cincinnati VA Medical Center	Cincinnati, OH
Dayton VA Medical Center	Dayton, OH
James A. Haley Veterans Hospital	Tampa, FL
James J. Peters VA Medical Center	Bronx, NY
Louis Stokes Cleveland VA Medical Center	Cleveland, OH
Malcom Randall VA Medical Center	Gainesville, FL
Miami VA Healthcare System	Miami, FL
Michael E. DeBakey VA Medical Center	Houston, TX
Minneapolis VA Medical Center	Minneapolis, MN
Phoenix VA Healthcare System	Phoenix, AZ
Portland VA Medical Center	Portland, OR
Salem VA Medical Center	Salem, VA
South Texas Veterans Healthcare System	San Antonio, TX
VA Ann Arbor Healthcare System	Ann Arbor, MI
VA Eastern Colorado Healthcare System	Denver, CO
VA Greater Los Angeles Healthcare System, West LA	Los Angeles, CA
VA Loma Linda Healthcare System	Loma Linda, CA
VA Long Beach Healthcare System	Long Beach, CA
VA North Texas Health Care System: Dallas VA Medical Center	Dallas, TX
VA Northern California Healthcare System, Sacramento	Mather, CA
VA Palo Alto Healthcare System	Palo Alto, CA
VA Salt Lake City Healthcare System	Salt Lake City, UT
VA St. Louis Healthcare System, John Cochran Division	St. Louis, MO
W.G. (Bill) Hefner VA Medical Center	Salisbury, NC
Washington DC VA Medical Center	Washington, DC
William S. Middleton Memorial Veterans Hospital	Madison, WI
CSP centers	
Massachusetts Veterans Epidemiology Research and Information Center (MAVERIC) CSP Coordinating Center	Boston, MA
CSP Clinical Research Pharmacy Coordinating Center (CSPCRPCC)	Albuquerque, NM

### Participants

VA-INTREPID will enroll and randomize a total of 880 study participants. The key inclusion criterion is a diagnosis of osteomyelitis in the diabetic foot, as defined by the International Working Group on the Diabetic Foot [18]. The key exclusion criteria are therapy with drugs that have critical interactions with rifampin, that either require empiric dose adjustment, or are considered absolutely contraindicated in combination with rifampin. The identity of the infecting organism(s) is not an inclusion or exclusion criterion. Full inclusion and exclusion criteria are listed in Table 3. Permission to approach patients who screen as eligible for the study will be obtained from clinical providers. Prior to enrollment, study personnel will explain all aspects of the study to potential participants and obtain informed consent in keeping with guidelines for human research subjects protection. Participants will be followed actively through the end of the second year after randomization or until death occurs, with the exceptions of those who withdraw consent early or who enroll in the last year of study recruitment. Participants will be encouraged at each study visit to complete the trial. If consent is withdrawn, study personnel will confirm with the participant whether they can continue reviewing the participant’s medical record until the end of the expected study participation. If a participant is lost to follow-up, regular review the medical record will continue until the end of the expected study participation. On average, study participants will be followed for 1.8 years through systematic review of medical records, and by study visits and phone calls. For participants who reach a primary endpoint of amputation, study drug, if still being administered, will be discontinued and continuing medical care will be determined by the treating providers. In such cases, participants will continue to be followed actively according to the study visit schedule. Participants who discontinue study drug treatment early due to an adverse event will also continue to be followed actively according to the study visit schedule.

**Table 3:** Study population

Inclusion Criteria:	
● Men and Women age ≥ 18 and ≤ 89 years	
● Diabetes Mellitus	
○ Defined either by: 1) use of oral hypoglycemic agents or insulin at the time of enrollment; or 2) a hemoglobin A1c (HgA1c) level within the past 90 days >6.5	
● Definite or probable osteomyelitis of the foot (DFO)	
○ Defined by the International Working Group on the Diabetic Foot (Table 1)	
● All planned debridement has been completed prior to randomization	
● A definitive course of backbone antibiotic treatment has been selected	
Exclusion Criteria:	
● Patient is unable to receive enteral medication	
● Patient is allergic to or intolerant of rifampin	
● Patient is taking a drug that has interactions with rifampin that would require either stoppage, substitution or an empiric dose modification that may place the patient at medical risk	
● Within 30 days of enrollment, patient is taking immunosuppressive medications to prevent rejection of an organ transplant or is receiving chemotherapy or molecularly targeted therapies for cancer	
● Patient is receiving antiretroviral therapy for HIV or antiviral medication for Hepatitis B or C	
● Enrollment in another trial of a therapeutic agent with a documented or suspected interaction with rifampin	
● Patient has an ALT >3 times the upper limit of normal for the site laboratory, or total bilirubin >2.5 times the upper limit of normal for the site laboratory; patient has Child-Pugh Class C Cirrhosis.	
● Patient has a baseline white blood cell count (WBC) <2000 cells/mm3 OR platelet count <50,000 cells/mm3 OR hemoglobin <8.0 g/dL.	
● Women of child-bearing potential (those with menses within the last year) with a positive serum pregnancy test.	
● Patient is believed unlikely to be able to complete the trial due to medical conditions such as metastatic cancer or end-stage organ failure	
● Patient is believed unlikely to complete the trial due to neurologic and psycho-behavioral disorders such as active substance abuse or dependence, disabling dementias or psychoses	
● Patient refuses or is clinically unable to undergo the recommended level of debridement	
● Patient’s prescribed backbone antibiotic therapy does not meet standard of care for either empirical treatment or culture-directed therapy	
● Indwelling hardware present in the foot, at the site of the index osteomyelitis	
● Treatment with antibacterial agents for infection at another site, where the duration of treatment is anticipated to be greater than 14 days	

### Management of Potential Drug-drug Interactions

The research team will screen study candidates for potentially contraindicated or interacting medications when taken with blinded study drug (rifampin or placebo) that would preclude enrollment. Patients will be excluded from the trial if they are 1) taking a medication that is considered contraindicated when combined with rifampin, or 2) taking a medication which would require a priori dose adjustment if rifampin was utilized, because blinding would preclude such dose adjustments. Table 4 contains a list of all excluded medications. After consent and immediately prior to randomization, the study team will again review the patients continued eligibility including any new contraindicated medications that, and confirm the patients continued willingness to participate. Finally, the research team will review concomitant medications while participants receive treatment with study drug to determine if a concomitant medication which would have precluded enrollment has been started and would require early discontinuation of study medication. The study participants and local research teams will be blinded to treatment assignment throughout the trial’s conduct. In instances where knowledge of the study treatment assignment would influence emergency medical treatment, unblinding may occur after consultation with a Study Chair and by obtaining information regarding treatment assignment from the CSPCRPCC.

**Table 4:** Excluded Concomitant Medications

Contraindicated	
Artemether	
Atazanavir	
Bocepravir	
Cobicistat	
Daclatasvir	
Darunavir	
Dasabuvir	
Delamanid	
Elbasvir	
Elvitegravir	
Fosamprenavir	
Grazoprevir	
Isovuconazonium	
Lopinavir	
Lurasidone	
Maraviroc	
Nelfinavir	
Ombitasvir	
Pariteprevir	
Praziquantel	
Ranolazine	
Rilpivirine	
Ritonavir	
Saquinavir	
Telaprevir	
Tipranivir	
Voriconazole	
Require Empiric Dose Adjustment	
Abiraterone	
Afatinib	
Amiodarone	
Aripiprazole	
Apixaban	
Cabozanitib	
Canaglifozin	
Clozapine	
Hormonal contraceptives	
Cyclosporine	
Dabigatran etexilate mesylate	
Desferasirox	
Digoxin	
Disopyramide	
Dolutegravir	
Dronedarone	
Edoxaban	
Efavirez	
Erlotinib	
Everolimus	
Exemestane	
Fosphenytoin	
Gefitinib	
Guanfacine	
Ibrutinib	
Imatinib	
Ixabepilone	
Mexilitene	
Lamotrigine	
Lapatinib	
Long acting opioids	
Phenytoin	
Propafenone	
Quetiapine	
Quinidine	
Raltegravir	
Rivaroxaban	
Temsirolimus	
Ticragrelor	
Vilazodone	
Vortioxetine	
Warfarin	

### Study intervention

Subjects will be randomly assigned to adjunctive rifampin or similar-appearing riboflavin placebo orally once daily for a six-week period. Each capsule of matching placebo will contain 12.5 mg of riboflavin for purposes of mimicking urine discoloration produced by rifampin.

The rifampin dose will be 600 mg orally daily, taken as two capsules of rifampin 300 mg once daily. If a subject experiences gastrointestinal intolerance on once daily dosing, the study drug may be administered as one capsule of rifampin 300 mg or placebo taken twice daily. Subjects will be informed that the study drug (either rifampin or the riboflavin placebo) may or may not cause a discoloration of their urine and other bodily fluids ranging from bright yellow to orange to orange-red.

Study drug therapy will be started immediately after randomization and will be prescribed for a treatment course totaling 84 capsules over a period of six calendar-weeks (42 days). Study drug administration will be discontinued: 1) at the time that a primary endpoint is reached; 2) at completion of protocol-defined therapy on Day 42; 3) if the participant experiences an adverse event that is considered to be at least possibly related to rifampin and that reaches grade 3 or 4 severity (Table 5); 4) if the LSI determines that continued study drug administration jeopardizes patient safety; 5) if the participant withdraws consent for the study; 6) if backbone antibiotic therapy is discontinued for clinical reasons earlier than initially planned, 7) if the participant requires a new concomitant medication that is listed as an exclusion criterion for study enrollment and requires this medication for a total course lasting more than 72 h.

**Table 5:** Toxicity Criteria [20]

	Mild (Grade 1)	Moderate (Grade 2)	Severe or Medically Significant but Not Immediately Life-Threatening (Grade 3)	Life-Threatening Consequences (Grade 4)
Liver Enzymes –either ALT, AST increase by factor	>ULN-3.0 x ULN	>3.0–5.0 x ULN	>5.0–20 x ULN	> 20 x ULN
Bilirubin	>ULN-1.5 x ULN	>1.5–3.0 x ULN	>3.0–10.0 x ULN	>10 x ULN
Creatinine – mg/dL Normal baseline	1.5 x ULN	>1.5–3.0 x ULN	>3.0–6.0 x ULN	> 6.0 x ULN
Creatinine – mg/dL Elevated baseline	1.5 x baseline	>1.5–3.0 x baseline	>3.0–6.0 x baseline	> 6.0 x baseline
Hemoglobin gm/dL	< LLN-10.0	< 10.0 to 8.0	< 8.0	Life-threatening, urgent intervention indicated
WBC Decrease - cell/mm^3^	LLN- 3000	2000 – 3000	1000 – 2000	< 1000
Platelets Decreased - cell/mm^3^	LLN - 75,000	50,000 - < 75,000	< 50,000–25,000	< 25,000

Commercially-available rifampin 300 mg capsules will be acquired by the CSPCRPCC and re-bottled into blinded packaging in accordance with current good manufacturing practices (cGMP). The CSPCRPCC will also manufacture matching placebo capsules containing 12.5 mg of riboflavin under cGMP conditions. The matching placebo will be similar in exterior appearance to the acquired rifampin 300 mg capsules and will be bottled into matching blinded packaging. Bottles of study medication will be labeled with unique bottle numbers to facilitate blinded administration.

### Backbone antibiotic therapy

Backbone antibiotic therapy will be selected by the local treatment team. The oral or intravenous backbone therapy selected by the treating physician will be communicated to the CSP coordinating center when the subject is enrolled to support stratification by route of administration of backbone therapy. Backbone therapy may be discontinued and replaced by alternative agents by the local treatment team in the event of drug intolerance, toxicity, hypersensitivity reaction, change in route of administration (e.g. switch from oxacillin to levofloxacin for MSSA), or recovery of microorganisms that are resistant to the selected agents or more effectively treated by a different agent.

### Surgical and podiatric management

Sites will be expected to follow the recommendations described in the Delphi consensus statement on surgical management of diabetic foot osteomyelitis [21]. Sites will be expected to utilize the most effective offloading method available [22]. Subjects will not be excluded for failure to comply with the recommended method of off-loading.

### Outcome measures

#### Primary outcome

The primary endpoint is amputation-free survival, ending with amputation or death from any cause. Amputation is defined as surgical treatment of osteomyelitis by removal or debridement of necrotic bone (all or part of a bone) from a lower extremity limb or digit on the ipsilateral side of the protocol-treated osteomyelitis. Debridement prior to randomization may include removal of bone. Because this debridement occurs early, prior to exposure to study drug or placebo, removal of bone at that time is not a study endpoint.

The amputation component of the primary endpoint for procedures at the site will be determined and documented by the Site Investigator’s review of all written operative notes and reports and surgical pathology reports within VA or outside medical facilities. The survival component of the primary endpoint will be determined by review of the medical record, review of death records, and telephone call to the phone number of record. All primary endpoints will be confirmed by the Site Investigator, and if requested, by final consultation with and confirmation by the Study Chair’s Office. The primary efficacy analysis will be on the intention-to-treat (ITT) population. The analysis will include all randomized subjects according to treatment assignment.

#### Secondary outcomes

Secondary outcomes include: 1) time from randomization to the occurrence of each component of the primary outcome 2) new courses of antibacterial therapy for ipsilateral foot infection during the first year after randomization, 3) quality of life measured by the 36-Item Short Form Health Survey (SF-36), 4) ambulatory status, 5) incidence of falls, 6) incidence of adverse events due either to direct drug toxicity or to drug-drug interactions, 7) remission of osteomyelitis at 12 months (defined as epithelialization of any overlying soft tissue defect and the absence of local signs and symptoms of inflammation), and 8) time to complete epithelialization of the wound.

### Adverse events

Participants will be assessed for potential rifampin toxicity every 2 weeks during the treatment course with study medication, as outlined in Fig. 1. Toxicity to study medication detected from the select laboratory studies will be graded according to Table 5. All serious adverse events and certain non-serious adverse events that occur after treatment initiation and before 6 weeks post-completion of study medication will be collected. For adverse events that do not result in a serious outcome, they will only be collected if the local site investigator considers the event to be at least possibly related to study medication. Additionally, reports of bodily fluid discoloration (unless it has caused the participant to seek medical care) and mild to moderate toxicities will not be collected as adverse events.

### Data collection and management

The MAVERIC CSPCC will manage clinical data and study documents using an Electronic Data Capture (EDC) and clinical trial management system (CTMS). The EDC system captures clinical data using electronic case report forms (CRFs), which are then stored at a central server location. Use of the EDC system allows site personnel to conduct data entry, review edit checks, and make updates to resolve discrepancies.

### Ethical considerations

The protocol and Informed Consent Form have been reviewed and approved by the Coordinating Center’s Human Rights Committee and by VA’s Central Institutional Review Board. Written informed consent will be obtained from all study participants consistent with the requirements of the Common Rule. An independent data monitoring committee (DMC) will meet semiannually to provide treatment effects monitoring during the trial’s conduct supplemented by real-time monitoring of safety events at the CSPCRPCC. The CSP Site Monitoring and Auditing Resource Team (SMART) will perform site and remote monitoring and auditing throughout the trial, with assistance from the Boston CSP Coordinating Center and the CSPCRPCC. Onsite monitoring visits will be conducted by SMART and will focus on assuring that study site personnel understand and follow the protocol and employ a risk-based approach to source document verification and source document review of original records. Each site will receive one onsite visit followed by additional onsite visits as needed based on any identified issues. Remote data review of critical data will also be routinely conducted by SMART throughout the trial. Finally, staff at the Boston CSP Coordinating Center will review Informed Consent Forms and regulatory documentation as well as generate reports from site data in order to asses site performance. The Food and Drug Administration has determined that VA-INTREPID is exempt from investigational new drug requirements.

### Biostatistical considerations

#### Sample size and statistical power considerations for the primary hypotheses

Based on data obtained from the Veterans Health Administration Corporate Data Warehouse, and published studies we hypothesized a relative reduction of 25% in the 2-year event rate with the use of rifampin as an adjunctive therapy compared with the use of adjunctive placebo. Data on the primary outcome measure will be analyzed by means of the two-sided log-rank test at a two-sided 5% significance level. The test has 90% power to detect a hazard ratio of 0.67 or lower with a total of 880 study participants, 440 per study arm. This allows for an interim analysis using O’Brian-Fleming [23] approach after half of the events in the trial have been observed and assumes that at most 6% of the study participants are lost to follow-up over the course of their participation into the study and before a study event is observed.

#### Randomization

After confirming eligibility, participants will be randomized in a 1:1 fashion to adjunctive rifampin or placebo by the research team using a centrally-administered interactive web response system (IWRS). The IWRS will also be used to facilitate blinded administration of study medication by providing the research team with a unique bottle number that contains study medication located on-site that corresponds to the participant’s assignment. To control for potential imbalance in randomization, both stratification and blocking will be employed. The randomization scheme, which will be generated by the study biostatistician and utilized by the IWRS when randomizing participants, will be stratified by participating site in addition to predominant route (oral or intravenous) of the clinician-prescribed backbone antibiotic regimen. Participants will be randomized to adjunctive therapy of rifampin or placebo within permuted random blocks.

### Statistical methods

The primary analysis will be performed according to the intention-to-treat (ITT) principle. Sensitivity analyses will be performed based on adherence to study drug during the six-week treatment phase by conducting the proposed analyses on the per-protocol (PP) set. The PP set will include participants adherent to the study medication. in the arm they were randomized to. A patient will be considered adherent if pill counts indicate that he/she took two-thirds or more of the dispensed 84 pills (i.e. 56 pills or more) of treatment medication during the 6 weeks after dispensation of the study medication. An additional sensitivity analysis will be carried out on a modified per-protocol (mPP) set that will account for participants who were so briefly on-study drug that the treatment was not likely to have had an effect. The mPP set will include in addition to patients in the PP set the patients who had their study medication withdrawn because of an outcome event and have taken their study medication for at least two-thirds of the indicated study drug prior to the outcome event. Adherence with study drug (rifampin or placebo) will be primarily assessed by pill count during the study visit at 6 weeks. The assessment of study drug adherence at the 6-week research visit will be used for the PP analysis. These sensitivity analyses will be considered as supplemental to the ITT analysis of the primary and secondary efficacy endpoints. Secondary outcomes will be analyzed using a Cox regression model, logistic regression analysis, or log rank testing, as appropriate.

### Data analysis of the primary endpoint

The primary analysis will be a time-to-event analysis with the use of the log-rank test based on intention-to-treat principles. Analytic reports will provide the hazard ratios and the 95% confidence interval about the hazard ratio. Kaplan-Meier curves will be used to represent estimates of the amputation-free survival distribution in the two intervention groups. Reports will also include estimates of event rates in the two treatment groups at 6 months, 1-year and 2-years of follow-up. Primary analyses will be followed by exploratory analyses, using Cox proportional hazards regression modeling, to account for the effects of baseline covariates on the primary outcome measure.

### Interim analysis

An interim analysis, considering stops for both superiority and futility, will be performed after approximately 50% of the planned total number of events has occurred (155 events of the anticipated 310 events). An O’Brien–Fleming stopping boundary for efficacy and futility will be used. Our calculations indicate that this will be achieved around month 28 into the study or after 685 subjects are enrolled. Based on the O’Brien Fleming boundary, at the interim analysis, it is recommended to stop for superiority if the two-sided *p*-value is < 0.0052 and the estimated Hazard Ratio comparing the risk of amputation or death between the rifampin and placebo is < 1, and we will reject for futility if the 2 sided p-value is < 0.0052 and the estimated Hazard Ratio comparing the risk of amputation or death between the rifampin and placebo is > 1. Additionally, we will confer with the Data Monitoring Committee (DMC) members and the program leadership for potential stopping guidelines based on findings from the interim analysis.

### Harms

Given the comorbidity expected in the study population, it is anticipated that a large number of adverse events (AEs) will be observed, most of which will not be related to the study intervention. For this reason, the study will only collect reports of all severe adverse events and those non-serious AEs that, in a site investigator’s clinical judgment, are at least possibly attributed to a study intervention and cannot be attributed to non-study intervention causes.

## Discussion

Adjunctive rifampin therapy is commonly employed in management of osteomyelitis in Europe, especially when *S. aureus* is identified [5, 6]. In contrast, data from the VA Corporate Data Warehouse showed that only 2% of cases of DFO were treated with rifampin. Physicians in North America may be dissuaded by the lack of an FDA indication for rifampin in osteomyelitis, or by concern for direct drug toxicity or drug interactions [24]. If the null hypothesis is rejected, including adjunctive rifampin with treatment of DFO in North America will be strongly supported, which should lead to a decrease in amputations and improved survival among patients with DFO. If the study shows no difference in outcomes with the addition of rifampin to backbone therapy, reconsideration of current management of DFO in Europe would be warranted. As newer agents with activity against bacteria in biofilms are developed [25], the demonstration of effectiveness of rifampin therapy will set the stage for new combination therapy approaches. The trial will provide safety data for rifampin in patients in an older age group with a high burden of comorbidity. The rich database provided by VA INTREPID will inform numerous aspects of DFO management including the impact of route of administration of backbone antimicrobial therapy, microbial etiology, role of vascular perfusion, glycemic control, effect of offloading modalities on outcomes, and the role of serum inflammatory markers in predicting outcomes.

Preliminary data obtained from the VHA Corporate Data Warehouse showed improved outcomes in patients with DFO treated with adjunctive rifampin, regardless of bone culture results. We therefore designed this study as a large, simple trial, including patients with DFO without regard to the identity of the infecting pathogen(s). Previous studies of rifampin adjunctive therapy for osteomyelitis focus on patients with staphylococcal infections and are therefore not generalizable to treatment of DFO as a whole [13]. Outcome data will be analyzed to determine whether use of rifampin should be broadly recommended or limited to a restricted range of organisms.

The diagnosis of DFO is most certain when bone biopsy shows positive cultures and histopathology, but these data are not available in up to 50% of cases of subsequently confirmed osteomyelitis [18]. Rather than limiting the study recruitment and the generalizability of the results to patients with definite DFO, we adopted the entry criteria for definite or probable DFO, according to the criteria of the International Working Group on the Diabetic Foot [18].

Studies of DFO commonly use wound healing as the primary outcome [26]. Given the high mortality observed in published studies of diabetic foot infections [27], and in our preliminary data, we chose to also include mortality in the primary outcome. Amputation free survival is an objective outcome, leaving little room for interpretation, which is appropriate for a large, simple trial. While most amputations will result from failure of treatment of the index infection, some amputations will occur due to biomechanical issues, or new infection that is not adjacent to the index osteomyelitis. These will be included in the primary outcome. A secondary outcome, ipsilateral amputation for the treatment of osteomyelitis related to the index osteomyelitis, will allow us to gather data that are more specific to the efficacy of the study intervention in treatment of infection. Wound healing at 1 year is an important, patient centered outcome, which we will also analyze as a secondary outcome.

Maintaining the integrity of the blind for rifampin posed unique challenges arising from the distinct physical appearance of rifampin, which appears as a red-brown crystalline powder, and arising from rifampin’s ability to discolor bodily fluids [28]. To mitigate the risk of unblinding and bias arising from knowledge of the treatment assignment by either patients or study personnel, the matching placebo will be manufactured such that it will be similar in exterior appearance to the rifampin product used in this trial. Riboflavin, which can also discolor urine, will be added to the matching placebo so that patients and the site research teams can be informed that both study medications discolor bodily fluids. The risk of information bias adversely affecting the trial’s internal validity will be further mitigated by employing a composite primary outcome with components that are readily detectable and ascertainable in the medical record and objectively evaluated.

Pharmacokinetic interactions between rifampin and other drugs are very common, limiting the number of patients who may be treated. Retrospective data suggest that 18% of Veterans with DFO had an active prescription for one of the common contraindicated medications. While many interactions can be managed by dose adjustment, some are considered contraindications to the use of rifampin. This trial will further elucidate the frequency of use of interacting drugs in this population, and the clinical impact of combination of rifampin with drugs that are considered to have a mild to moderate interaction.

VA-INTREPID is a large, simple trial of a readily available, inexpensive medication that is commonly employed in Europe. Our preliminary data suggest that rifampin may improve amputation free survival in patients with DFO. A limitation of the design is the inability to identify a masking agent that perfectly mimics the effect of rifampin on body fluids. The choice of riboflavin was driven by its safety, as compared to other possibilities, e.g. pyridium. Inclusion of patients who have pathogens with less sensitivity to rifampin than *S. aureus* may increase the risk of a Type 2 error. However, our preliminary data suggested that patients with and without cultures positive for *S. aureus* had similar benefit from rifampin. Furthermore, secondary analyses of the impact of bacteriology on the primary outcome could help to detect an effect. The strengths of the design include the use of a consensus case definition for DFO, objectively evaluated primary outcomes, stringent power analysis, and a multicenter design.

## Data Availability

Digital data underlying primary scientific publications from this study will be held as part of a data sharing resource maintained by the Cooperative Studies Program (CSP). Study data held for this purpose may include data, data content, format, and organization. The data may contain but are not limited to individually identifiable information, other protected health information, and study codes. The data may be available to the public and other VA and non-VA researchers under certain conditions and consistent with the informed consent and CSP policy which prioritize protecting subjects’ privacy and confidentiality to the fullest extent possible. It is the policy of the CSP that outcome data will not be revealed to the participating investigators until the study is completed. This policy safeguards against possible biases affecting the data collection. The presentation or publication of any or all data collected by participating investigators on patients entered into the VA Cooperative Study is under the direct control of the study’s Executive Committee. No individual participating investigator has any inherent right to perform analyses or interpretations or to make public presentations or seek publication of any or all of the data other than under the auspices and approval of the Executive Committee.

## Abbreviations

AE: Adverse Event
ALT: Alanine Aminotransferase
AST: Aspartate Aminotransferase
cGMP: Current Good Manufacturing Practices
CRF: Case Report Form
CRPCC: Clinical Research Pharmacy Coordinating Center
CSP: Cooperative Studies Program
CSPCC: Cooperative Studies Program Coordinating Center
CTMS: Clinical Trial Management System
DFO: Diabetic Foot Osteomyelitis
DMC: Data Monitoring Committee
EDC: Electronic Data Capture
FDA: Food and Drug Administration
GCP: Good Clinical Practice
HgA1c: Hemoglobin A1c
HIV: Human Immunodeficiency Virus
ICMJE: International Committee of Medical Journal Editors
INTREPID: Investigation of Rifampin to Reduce Pedal Amputations for Osteomyelitis in Diabetics
ITT: Intention to Treat
IWRS: Interactive Web Response System
LLN: Lower Limit of Normal
MAVERIC: Massachusetts Veterans Epidemiology Research and Information Center
mPP: Modified Per-Protocol
MRI: Magnetic Resonance Imaging
NHANES: National Health and Nutritional Examination Survey
PP: Per-Protocol
SF-36: Short Form-36
SMART: Site Monitoring, Auditing, and Resource Team
TCpO2: Transcutaneous Oxygen Pressure
ULN: Upper Limit of Normal
VA: Veterans Affairs
VHA: Veterans Health Administration
WBC: White Blood Cell count
